# Population genetic structure and connectivity of the seagrass *Thalassia hemprichii* in the Western Indian Ocean is influenced by predominant ocean currents

**DOI:** 10.1002/ece3.5420

**Published:** 2019-07-23

**Authors:** Marlene Jahnke, Martin Gullström, Josefine Larsson, Maria E. Asplund, Said Mgeleka, Mathew Ogalo Silas, Arielle Hoamby, Jamal Mahafina, Lina Mtwana Nordlund

**Affiliations:** ^1^ Department of Marine Sciences—Tjärnö University of Gothenburg Strömstad Sweden; ^2^ Groningen Institute for Evolutionary Life Sciences, Section: Ecology and Evolutionary Genomics in Nature (GREEN) University of Groningen Groningen The Netherlands; ^3^ Department of Ecology, Environment and Plant Sciences Stockholm University Stockholm Sweden; ^4^ Department of Biological and Environmental Sciences University of Gothenburg Gothenburg Sweden; ^5^ School of Natural Science, Technology and Environmental Studies Södertörn University Stockholm Sweden; ^6^ The Lovén Centre University of Gothenburg Gothenburg Sweden; ^7^ Tanzania Fisheries Research Institute (TAFIRI) Dar es Salaam Tanzania; ^8^ Institut Halieutique et des Science Marine Toliara (IH.SM) Toliara Madagascar; ^9^ Department of Earth Sciences Uppsala University Visby Sweden

**Keywords:** coastal conservation, connectivity, dispersal, gene flow, genetic structure, microsatellite, ocean current, population genetics, seagrass, Western Indian Ocean

## Abstract

This study is the first large‐scale genetic population study of a widespread climax species of seagrass, *Thalassia hemprichii*, in the Western Indian Ocean (WIO). The aim was to understand genetic population structure and connectivity of *T. hemprichii* in relation to hydrodynamic features. We genotyped 205 individual seagrass shoots from 11 sites across the WIO, spanning over a distance of ~2,700 km, with twelve microsatellite markers. Seagrass shoots were sampled in Kenya, Tanzania (mainland and Zanzibar), Mozambique, and Madagascar: 4–26°S and 33–48°E. We assessed clonality and visualized genetic diversity and genetic population differentiation. We used Bayesian clustering approaches (TESS) to trace spatial ancestry of populations and used directional migration rates (DivMigrate) to identify sources of gene flow. We identified four genetically differentiated groups: (a) samples from the Zanzibar channel; (b) Mozambique; (c) Madagascar; and (d) the east coast of Zanzibar and Kenya. Significant pairwise population genetic differentiation was found among many sites. Isolation by distance was detected for the estimated magnitude of divergence (*D*_EST_), but the three predominant ocean current systems (i.e., East African Coastal Current, North East Madagascar Current, and the South Equatorial Current) also determine genetic connectivity and genetic structure. Directional migration rates indicate that Madagascar acts as an important source population. Overall, clonality was moderate to high with large differences among sampling sites, indicating relatively low, but spatially variable sexual reproduction rates. The strongest genetic break was identified for three sites in the Zanzibar channel. Although isolation by distance is present, this study suggests that the three regionally predominant ocean current systems (i.e., East African Coastal Current, North East Madagascar Current, and the South Equatorial Current) rather than distance determine genetic connectivity and structure of *T. hemprichii* in the WIO. If the goal is to maintain genetic connectivity of *T. hemprichii* within the WIO, conservation planning and implementation of marine protection should be considered at the regional scale—across national borders.

## 1 INTRODUCTION

Understanding population genetic structure, long‐distance dispersal and connectivity patterns of organisms facilitates conservation actions in spatially widespread coastal seascapes (Jones et al., 2009). Population genetics may act as a powerful tool for resource management planners to understand the genetic connectivity between populations, which in turn may have implications for decisions regarding number, sizes, and locations of protected areas (Palumbi, 2003; Waycott et al., 2009). Furthermore, genetic studies can also provide estimates of genetic diversity, an important factor for an organism's adaptive capacity to survive in a changing environment (Smith & Bernatchez, 2008; Vandergast, Bohonak, Hathaway, Boys, & Fisher, 2008).

Seagrasses are marine flowering plants forming habitats (i.e., seagrass meadows) in coastal areas around the world and provide a wide variety of ecosystem services, such as habitat and nursery grounds for fish and invertebrates, carbon sequestration, and improving water quality (Angelini, Altieri, Silliman, & Bertness, 2011; Heck, Hays, & Orth, 2003; Nordlund, Koch, Barbier, & Creed, 2016). During the last decades, seagrass meadows have experienced a substantial decline (Orth et al., 2006; Waycott et al., 2009) and information regarding genetic structure and variability of meadows could provide an important link for coastal management actions. There are a few previous large‐scale assessments of seagrass population genetics worldwide (see Arriesgado et al., 2015, Hernawan et al., 2016; Jahnke et al., 2018, Sinclair et al., 2016; Triest et al., 2018; van Dijk, Mellors, & Waycott, 2014, Wainwright, Arlyza, & Karl, 2018 for recent examples). While in the Western Indian Ocean (WIO) we are only aware of one local assessment of the seagrass *Thalassodendron ciliatum* from 2001 in southern Mozambique using RAPD markers (Bandeira & Nilsson, 2001) and one recent study about the seagrass *Zostera capensis* sampled along the South African coast, and in one bay in Mozambique and one bay in Kenya indicating the presence of two population clusters broadly corresponding to populations on the west and east coasts of Africa (Phair, Toonen, Knapp, & Heyden, 2019).

The seagrass *Thalassia hemprichii* (Ehrenberg) Ascherson is widely distributed in the Indo‐Pacific (Green & Short, 2003). It is one of the most common seagrass species in the WIO (Bandeira & Björk, 2001; Gullström et al., 2002), a biogeographic sub‐region of the Indian Ocean stretching on a latitudinal scale from Somalia to the east coast of South Africa (Obura, 2012). *T. hemprichii* reproduces sexually by seeds and asexually by rhizome growth. The extent of each component of reproduction has important effects on local population demographics, dispersal, biogeography, and genetic diversity. Seed banks, in terms of long‐term survival of buried seeds, play an important role in the persistence of some seagrass species, but seem to be absent in *T. hemprichii* (Rollon, Vermaat, & Nacorda, 2003). In terms of long‐distance dispersal, positively buoyant shoots with attached rhizomes or seedling have the highest potential for long‐distance dispersal in *Thalassia* spp. (Kendrick et al., 2012; Wu, Chen, & Soong, 2016). In the west Pacific, adult plants of *T. hemprichii* have been shown to be able to float for months and still remain alive and potentially able to colonize new areas (Wu et al., 2016). Seeds seem to sink within 24 hr and fruits, while having a potential to float for about a month and at distances of dozens to hundreds of kilometers, do not seem to contain seeds that can germinate after long‐time floatation (van Dijk, Tussenbroek, Jiménez‐Durán, Márquez‐Guzmán, & Ouborg, 2009; Lacap, Vermaat, Rollon, & Nacorda, 2002; Wu et al., 2016). The fruits and seeds do not seem to survive the passage through the birds’ digestive tract (Wu et al., 2016). Therefore, long‐distance dispersal seems to be primarily dependent on buoyant shoots (and to a lesser degree on fruits) that move passively by currents (Thiel & Gutow, 2005). Hence, oceanographic features are expected to strongly shape the directionality of dispersal and/or to act as barriers for *T. hemprichii*.

**Figure 1:**
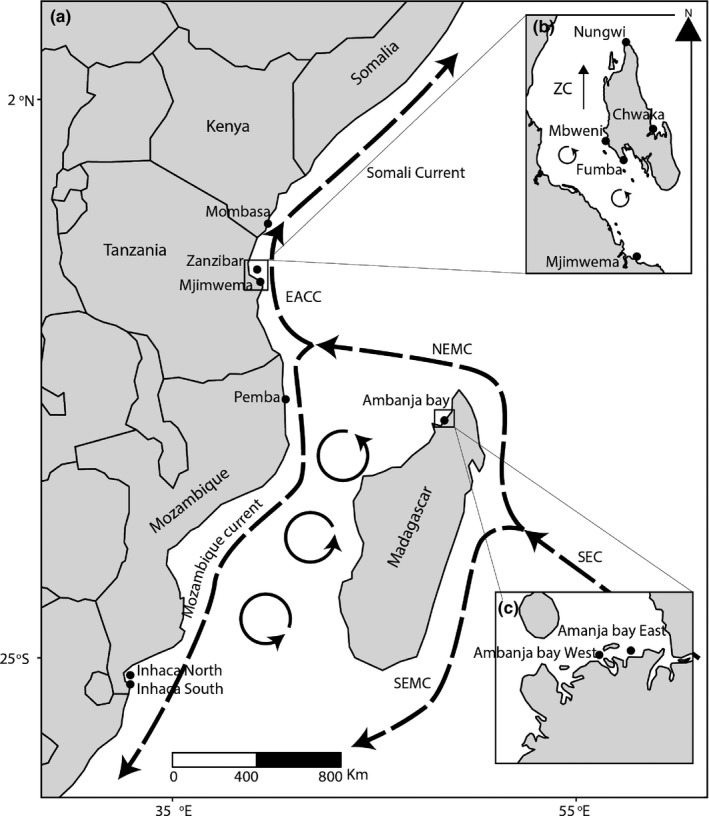
Map of sampling sites (indicated with black dots) in the WIO. Bold dashed lines are major current systems, while bold cycled lines show eddies. The major currents are the EACC (East African Coast Current), the NEMC (North East Madagascar Current), the SEMC (South East Madagascar Current), and the SEC (South Equatorial Current). ZC Zanzibar Channel. Acronyms (codes) for sampling sites are shown in Table 1

Major currents in the WIO region are driven by the east‐west flow of the South Equatorial Current (SEC) that carries water toward Madagascar, where it splits up into the North East Madagascar Current (NEMC) continuing toward the southern coast of Tanzania and the northern coast of Mozambique (Obura et al., 2018; Richmond, 2002) and the South East Madagascar Current (SEMC) continuing south along the east coast of Madagascar. Where the NEMC reaches the coast of southern Tanzania and northern Mozambique, this major current system splits into two major currents, that is, the East African Coastal Current (EACC) that flows northwards along the coasts of Tanzania and Kenya and the Mozambique current system that flows south along the coast of Mozambique creating complex eddies in the Mozambique Channel (Figure 1; Benny, 2002; Obura et al., 2018; Richmond, 2002).

In general, there is a gap of knowledge on marine genetic patterns in the WIO region (Ridgway & Sampayo, 2005), but see for example studies on corals (Souter, Henriksson, Olsson, & Grahn, 2009; van der Ven et al., 2016), spiny lobster (Singh, Groeneveld, Hart‐Davis, Backeberg, & Willows‐Munro, 2018), and fish (Muths, Gouws, Mwale, Tessier, & Bourjea, 2012; Visram et al., 2010). The available population genetic data indicate that the WIO is a unique genetic sub‐region of the Indian Ocean (Ridgway & Sampayo, 2005). This makes regional population genetic studies highly valuable for management initiatives in the WIO (Ridgway & Sampayo, 2005).

The main aim of the current study was to reveal the genetic population structure, dispersal patterns, and connectivity of the common and important habitat forming seagrass *T. hemprichii* in the WIO region, with focus on the coasts of Kenya, Tanzania (mainland and Zanzibar), Mozambique, and Madagascar. Since dispersal of both shoots and fruits of *T. hemprichii* mainly occurs by passive rafting, we hypothesized that genetic population structure in the region is shaped by the South Equatorial, the North East Madagascar, and the East African Coastal currents. The findings are used to discuss how to improve seagrass management in the WIO region to sustain healthy seagrass populations with high resilience to environmental change.

## 2 MATERIAL AND METHODS

### 2.1 Study area and field sampling

The WIO covers about 40 degrees of latitude and hosts a high level of marine biodiversity (from Somalia in the north to eastern South Africa in the south). The climate and pattern (and strength) of currents in the WIO are complex and strongly influenced by the monsoonal circulation (Figure 1). Throughout the SE monsoon season (March to October), the EACC is speeded up by the south‐easterly trade winds and consequently, the current speed reaches ca. 1.5–2 ms^−1^. During the NE monsoon season (October–March), the current is slowed down by the north‐easterly trade winds and as a consequence the current speed is lowered to about 0.5 ms^−1^ (McClanahan, 1988; Mahongo & Shaghude, 2014).

Field sampling was conducted between 2011 and 2017 in seagrass meadows dominated by *T. hemprichii*. The seagrass meadows are often patchy and where possible samples were collected from different patches. Since a large area was sampled in each site, and *T. hemprichii* often mixes with other species, there was no site that was fully monospecific. None of the sampling sites were located in conservation areas. At each site, 15–20 individual shoots of *T. hemprichii* were randomly collected by snorkeling or walking during low tide at a distance of 10–150 m apart to largely avoid sampling of genetically identical clones. Commonly, the distance between samples was 50–100 m. This resulted in the collection of 205 sampling units at 11 locations in Kenya, Tanzania (mainland and Zanzibar), Mozambique, and Madagascar, at pairwise distances between sampling sites of approximately 15–2,700 km (Table 1, Figure 1). One leaf per shoot and one piece of rhizome were cleaned of epiphytes and dried, and stored in silica crystals or frozen in 70% ethanol until DNA extraction.

**Table 1:** Genetic diversity of *Thalassia hemprichii* at 11 locations in the Western Indian Ocean

Country	Site	Code	Latitude	Longitude	*N*_s_	*N*_all_	MLGs	*R*	*A*_6_	sd_A	*H*_O_ (*SE*)	*H*_E_ (*SE*)	*F*_IS_ (*SE*)	% PL
Mozambique	Inhaca South	ZIS	26°02′08.0″S	32°55′56.2″E	20	20	16	0.79	1.49	0.03	0.06 (0.06)	0.21 (0.08)	0.77 (0.15)	42
Mozambique	Inhaca North	ZIN	25°49′43.3″S	32°54′50.0″E	20	20	6	0.26	1.5	0	0.08 (0.10)	0.22 (0.08)	0.67 (0.24)	50
Mozambique	Pemba	ZP	12°58′03.9″S	40°32′50.7″E	20	19	16	0.83	1.59	0.13	0.07 (0.04)	0.20 (0.06)	0.53 (0.14)	83
Tanzania ‐ Zanzibar	Fumba	TZF	6°19′20.8″S	39°17′20.5″E	20	20	13	0.63	1.56	0.07	0.09 (0.03)	0.13 (0.05)	0.26 (0.13)	75
Tanzania ‐ Zanzibar	Mbweni	TZM	6°13′25.7″S	39°11′49.2″E	20	18	10	0.53	1.28	0.06	0.05 (0.03)	0.09 (0.05)	0.54 (0.12)	25
Tanzania ‐ Zanzibar	Chwaka	TZC	6°09′44.14″S	39°26′29.14″E	20	20	19	0.95	1.59	0.05	0.06 (0.06)	0.22 (0.08)	0.79 (0.14)	42
Tanzania ‐ Zanzibar	Nungwi	TZN	5°43′02.22″S	39°18′06.80″E	20	20	15	0.74	1.45	0.06	0.04 (0.03)	0.15 (0.06)	0.75 (0.11)	42
Tanzania‐Mainland	Mjimwema	TM	6°49′23.2″S	39°20′59.9″E	15	7	6	0.83	1.33	0	0.08 (0.06)	0.13 (0.06)	0.49 (0.17)	33
Kenya	Mombasa	KM	4°00′41.54″S	39°43′43.27″E	20	19	17	0.89	1.59	0.13	0.06 (0.04)	0.23 (0.07)	0.76 (0.09)	58
Madagascar	Ambanja bay East	MAE	13°31′4.28″S	48°27′53.97″E	15	15	6	0.36	1.33	0	0.03 (0.03)	0.12 (0.05)	0.70 (0.17)	33
Madagascar	Ambanja bay West	MAW	13°31′23.88″S	48°25′32.34″E	15	14	6	0.38	1.17	0	0.01 (0.01)	0.05 (0.04)	0.45 (0.22)	17

Note

The 205 individuals sampled at the coasts of Kenya, Tanzania (mainland and Zanzibar), Mozambique, and Madagascar were assessed with 12 microsatellites. After the population codes are the geographical coordinates (latitude and longitude), the number of samples (*N*_S_), the number of individuals for which all 12 loci were successfully amplified (*N*_all_), the number of multilocus genotypes (MLG), genotypic richness (*R*), allelic richness standardized to 6 genotypes (*A*_6_), the standard deviation of A_6_ (sd_A), observed heterozygosity (*H*_O_), expected heterozygosity (*H*_E_), the fixation index (*F*) including the standard error (*SE*), and the percentage of polymorphic loci in each population (% PL).

### 2.2 DNA extraction and microsatellite amplification

We extracted DNA from ca. 20 mg of silica‐gel dried leaf tissue or from ethanol‐preserved rhizome tissue using modified CTAB protocols for silica‐preserved samples (Hoarau, Coyer, Stam, & Olsen, 2007) and ethanol‐preserved samples (Zuccarello & Lokhorst, 2005). We then amplified the samples with thirteen microsatellites as suggested by van Dijk et al. (2014), although only twelve markers amplified successfully. We combined the microsatellites in two different multiplexes in 96‐well plates, as suggested by van Dijk et al. (2014), and ran all PCRs in 6.2 μl reaction with Qiagen Type‐IT^®^. The temperature profiles were as follows: 95°C for 5 min, 30 cycles of 95°C for 30 s, 56°C for 1 min 30 s and 72°C for 30 s, with a final extension step of 60°C for 30 min (see Appendices S1 and S2 for details on primer sequences and PCR reagent concentrations).

### 2.3 Scoring and data quality checks

Fragment size analysis was performed with undiluted PCR products using an Applied Biosystems 3730 DNA Analyser with a 350 ROX internal size standard added to each well. The fragments were scored with PeakScanner^®^. Samples with an unclear allele pattern were re‐amplified and re‐genotyped. We did not succeed in amplifying all individuals at all loci, and individuals with missing data were removed.

When working with partially clonal organisms, it is important to distinguish between unique genotypes and clonal replicates, and we used RClone (Bailleul, Stoeckel, & Arnaud‐Haond, 2016) in R 3.3.1 (R Development Core Team, 2014) to identify multilocus genotypes (*MLG*s), that is clones. As most downstream analyses assume microsatellite loci to be independent from each other (i.e., not in linkage) and to be in Hardy–Weinberg equilibrium (HWE), we tested for HWE at each locus and across all loci in each population with Genepop 4.2 (Raymond & Rousset, 1995) using 100 batches and 1,000 iterations per batch, and applying Bonferroni corrections, and for Linkage Disequilibrium (LD), a modification of the standard methods, described by Agapow and Burt (2001) in the R package *poppr* (Kamvar, Tabima, & Grünwald, 2014), was applied using 1,000 permutations.

Loci with null alleles (i.e., not amplified allele copies) need to be removed from the dataset since they cause genotyping errors when one of the allele copies fails to be amplified by the PCR, which leads to missing genotypes and the incorrect assignment of homozygotes. To detect null alleles, we used the software MicroDrop (Wang & Rosenberg, 2012) with 10,000 permutations and 100 replicates. Furthermore, molecular markers used to assess connectivity should not be under selection, and therefore it is recommended to always test for outlier loci before estimating population genetic parameters (Luikart et al., 2003; Selkoe & Toonen; 2006; Gagnaire et al., 2015). To test if any of our loci deviate significantly from expectation under neutrality, we ran BayeScan (Foll & Gaggiotti, 2008) with default settings and used the R script provided by Foll and Gaggiotti (2008) to identify any loci showing signs of selection.

### 2.4 Genetic diversity and population differentiation

To estimate genetic diversity (genetic variation within each sampling site) and population differentiation (genetic difference between sampling sites) several calculations were made. Genotypic richness (*R*), a measure of the amount of clonal growth, was calculated with the formula (MLG − 1)/(*N* − 1), which relates the number of MLGs to the number of ramets (*N*) (Dorken & Eckert, 2001). The genetic diversity estimate of heterozygosity was calculated in GenAlEx 6.5 (Peakall & Smouse, 2012) and allelic richness, standardized to the same number of MLGs, with standArich (http://alberto-lab.blogspot.nl/p/code.html#!/p/code.html) in R 2.15.3. We estimated genetic differentiation by calculating pairwise Weir & Cockerham *F*_ST_ among populations (Weir & Cockerham, 1984) and the unbiased estimator of Jost *D*_EST_ (Jost, 2008) and *G*_ST_′ (Hedrick, 2005) with the *diveRsity* package (Keenan, McGinnity, Cross, Crozier, & Prodöhl, 2013) in R 3.2.2 using 1,000 bootstrap replicates to test for the significance of pairwise comparisons. To summarize the genetic structure present in our dataset, we used PCA as implemented in *adegenet 2.0.1* (Jombart, 2008) in R 3.3.2.

In order to detect spatial genetic structure in the WIO, we used Bayesian clustering algorithms implemented in STRUCTURE (Pritchard, Stephens, & Donnelly, 2000) and TESS 2.3 (Chen, Durand, Forbes, & François, 2007). Both programs perform Markov Chain Monte Carlo simulations with the aim to find the most probable number of genetic clusters under Hardy–Weinberg equilibrium. As we had geographic information only at the population level, we used TESS to calculate slightly adapted geographic coordinates for each individual. We then run TESS with the CAR admixture model, which assumes spatial autocorrelation of genetic differentiation, using the default value of 0.6 for the strength of the autocorrelation. We run TESS for an assumed number of clusters, *K*_max_ = 2–12 using a burn‐in of 10,000 sweeps followed by 25,000 sweeps, with 100 independent runs conducted for each *K*_max_. We then used the average deviance information criterion for each value of *K*_max_ to evaluate the most likely number of genetic clusters. For visualizing clusters and for post‐processing of TESS outputs, we used *pophelper* (Francis, 2016) with CLUMPP 1.1.2 (Jakobsson & Rosenberg, 2007) in R 3.2.2.

### 2.5 Directional migration rates and bottlenecks

To calculate directional migration rates among sampling sites, we used divMigrate‐online (https://popgen.shinyapps.io/divMigrate-online/) based on the genetic distance measures of *G*_ST_ (Sundqvist, Keenan, Zackrisson, Prodohl, & Kleinhans, 2016). To identify first‐generation migrants (individuals with different allele frequencies than the population where they were sampled), we also used an assignment test implemented in GENECLASS2 (Piry et al., 2004) with the exclusion method. We investigated local population dynamics and the potential presence of genetic bottlenecks with the program Bottleneck 1.2.02 (Cornuet & Luikart, 1996) assuming a step‐wise mutation model (SMM) and the two‐phased model of mutation (TPM) and using 10,000 replications. We only conducted this test at sites with more than 10 *MLG*s.

### 2.6 Isolation by distance

Isolation by distance (IBD) is the relationship of genetic similarity between sites over a geographic distance. The model tests the assumption that the further apart two sites are from each other geographically, the more genetically different they should be. To test for IBD, we correlated the genetic distance matrices with “sea distance” (geographic distance among sampling sites without crossing land). This measure was calculated with the R package *marmap* (Pante & Simon‐Bouhet, 2013). Subsequently, we carried out a Mantel test using the R package *ncf* (Bjornstad, 2009) in R 3.2.2 and resampling of the matrices was performed (100,000 times).

## 3 RESULTS

### 3.1 Genetic data quality control

Clonality varied among sites and we identified 6–19 genotypes per population (Table 1), resulting in 130 genets (out of 205 ramets) that were used for all further analyses. ZIN in Mozambique and the two Madagascan sites (MAW and MAE) exhibited the highest levels of clonality (lowest *R*‐values; Table 1). Several clones were shared among sites. In general, microsatellites were only lowly polymorphic in our African *T. hemprichii* samples (Table 1), especially compared to a recent Indo‐Australian study of *T. hemprichii* that used the same microsatellites (Hernawan et al., 2016). After quality control, all further analyses were based on the full set of 12 microsatellites 27% HWE tests per population and locus were significant and evidence of significant deviation from LD at one site only [ZIS] solely driven by the linkage across the loci TH66 and TH73 with an extremely high rd value of 1. No locus showed frequencies of null alleles above 10% and no locus showed signs of selection.

### 3.2 Genetic diversity and differentiation

Genetic diversity was generally low. The number of alleles per locus ranged from 1 (monomorphic) to 7 (TH07: 2, THH‐5:2, THH‐15:4, TH37: 3, TH66: 3, TH73: 3, THH‐34:3, TH34: 7, TH43: 2, TH52: 3, THH‐41:1, THH‐3:3). There were eleven rare alleles in our dataset (defined as occurring less than three times), which were spread among nine loci. Of these, only three were not previously recorded (by Hernawan et al., 2016). Standardized allelic richness (*A*_6_) across all loci ranged from 1.17 to 1.59, and the observed heterozygosity *H*_O_ was very low, ranging from 0.014 to 0.09, while expected heterozygosity *H*_E_ ranged from 0.054 to 0.234 (Table 1).

**Figure 2:**
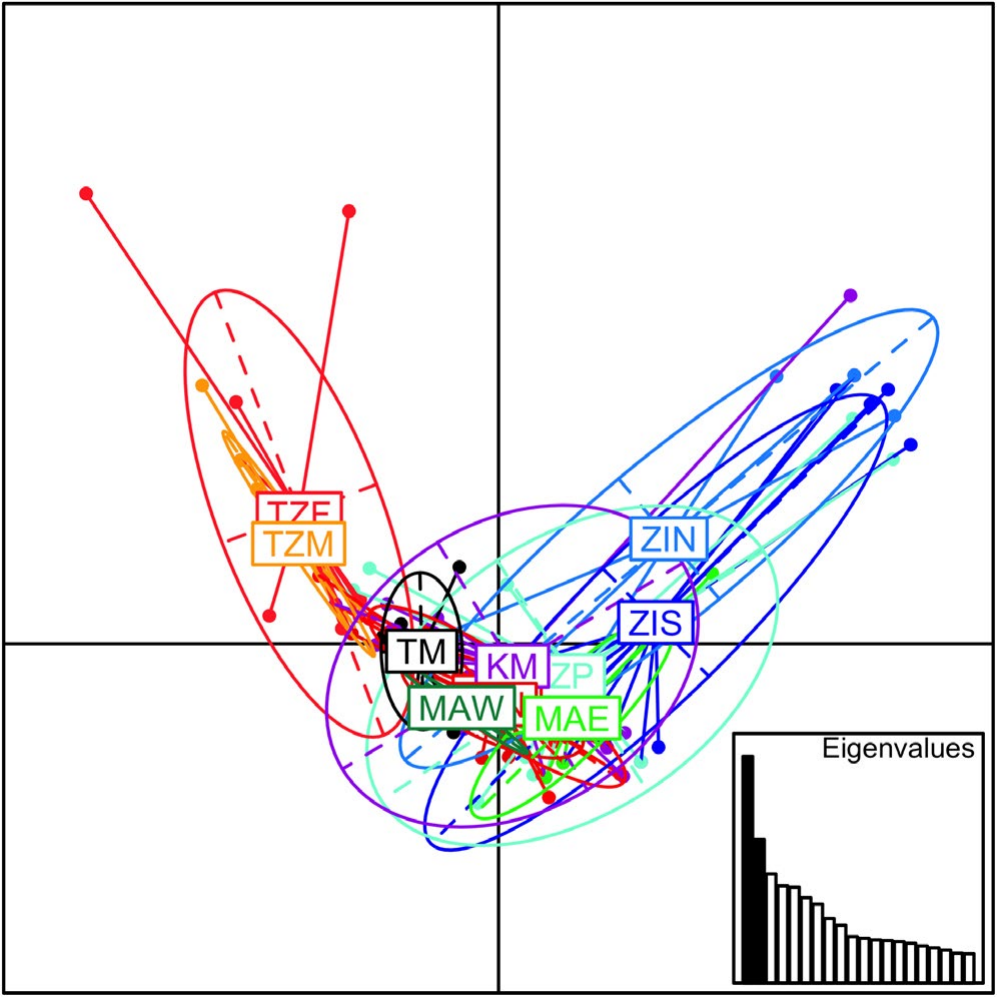
The PCA analysis of the 11 *Thalassia hemprichii* populations in the Western Indian Ocean shows a horseshoe pattern of differentiation on the first two axes. Samples coming from the four sites in Zanzibar (starting with TZ) are colored in shades of red, the Tanzanian mainland site (TM) in black, the three sites from Mozambique (starting with Z) in shades of blue, the two sites from Madagascar (starting with M) in shades of green, and the Kenyan sampling site (KM) in purple. Site acronyms as in Table 1 and Figure 1; the inset plot shows the eigenvalues of the first two principal components, which were used in this analysis

The genetic differentiation estimator *F*_ST_ (Weir & Cockerham, 1984) ranged between −0.05 and 0.64, and two‐thirds of the pairwise comparisons were significant (*p* < 0.05, Appendix S3). *G*_ST_′ and *D*_EST_ showed similar patterns of genetic differentiation and significance. The PCA showed little differentiation among most sampling sites with a horseshoe‐like pattern of few sites being differentiated on the first two axes (Figure 2; explaining 26% of the variance), and a similar pattern on the third axis (not shown). Such a pattern is expected under scenarios where genetic distance increase with geographic distance (Frichot, Schoville, Bouchard, & François, 2012). The most differentiated sampling sites were those from Zanzibar (TZM and TZF) and Mozambique (ZIN and ZIS) (Figure 2).

**Figure 3:**
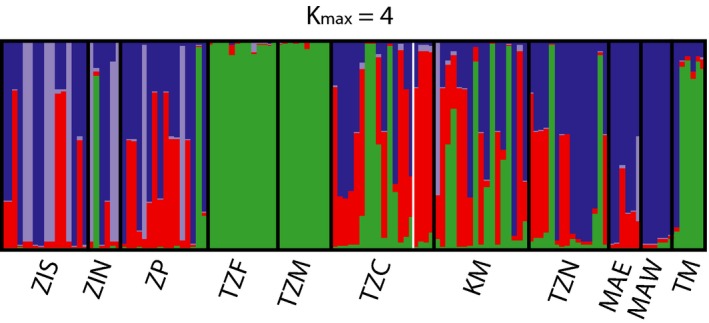
TESS clustering analysis of the 11 *Thalassia hemprichii* populations in the Western Indian Ocean. The TESS plot is shown for the most likely number of clusters (according to the deviance information criterion of averaged runs) and plots for other *K_max_* can be found in Appendix S6. Within each plot, each vertical bar represents an individual belonging to the sampling location indicated under the *x*‐axis, clusters are color coded, and the *y*‐axis of each plot shows the proportion of the genotype belonging to each cluster. Site acronyms as in Table 1 and Figure 1

The TESS cluster analysis suggested that our data could be divided into four genetic clusters (*K_max_* = 4, see Appendix S5). The four major genetic regions consist of the following: (a) samples from the Zanzibar channel (TZF, TZM, and TM; green cluster); (b) samples from Mozambique (ZIS, ZIN, and ZP; all have individuals belonging to a light blue cluster); (c) samples from Madagascar (MAE and MAW; individuals mostly belonging to the blue cluster); and (d samples from the east coast of Zanzibar (TZC and TZN) and Kenya (KM) which are admixed between clusters (Figure 3). For a higher number of clusters, no additional clear grouping was evident (see Appendix S6).

### 3.3 Directional migration rates

**Figure 4:**
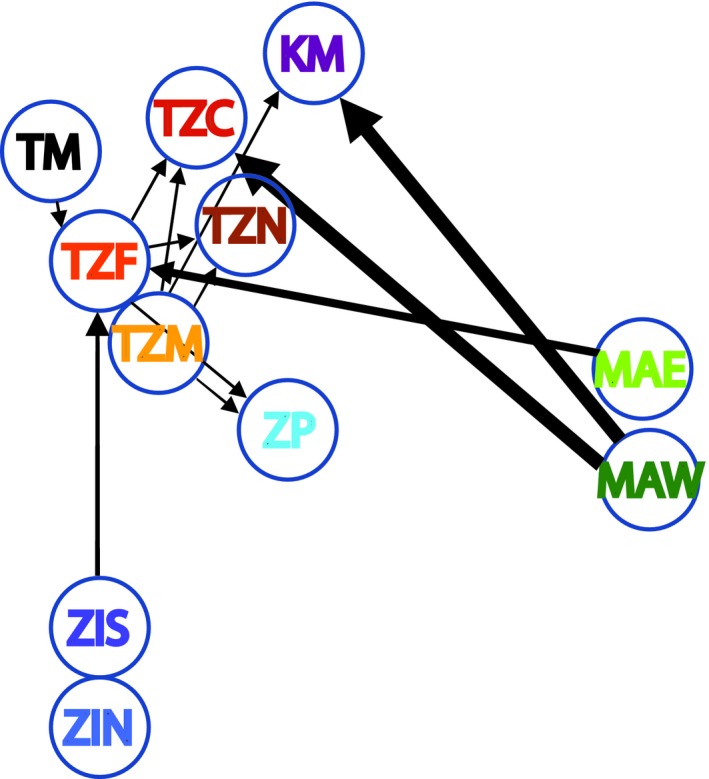
Asymmetrical migration rates of the 11 *Thalassia hemprichii* populations in the Western Indian Ocean calculated with DivMigrate and based on G_ST_. Acronyms are shown in Table 1 and letter coloring is as shown in Figure 2. Sites in the network are placed according to their approximate geographical location. Thick arrows present migration of 0.02, while the thinner arrows represent migration at levels of 0.001–0.006

We detected asymmetric migration (Figure 4) and not surprisingly, most gene flow was detected among the five geographically close sites in Tanzania (all starting with T). Highest gene flow was detected from the Madagascan site MAW to TZC on Zanzibar and the Kenyan site KM. The two sampling sites in the south of Mozambique (ZIS and ZIN) are most isolated (Figure 4).

The additional assignment test identified seven first‐ or second‐generation migrants, of which two could be assigned to other sampling sites (Table 2). The immigrant at the site ZP in Mozambique is predicted to come from either TZF or TZM (Zanzibar), as also suggested by the divMigrate analysis (Figure 4). The immigrant at the site TZN in Zanzibar is suggested to come from the site TZC in Zanzibar or the Kenyan site KM. This scenario is not supported by the divMigrate analysis, but all three populations come from the same cluster identified by TESS (Table 2, Figure 3).

**Table 2:** Assignment test of *Thalassia hemprichii* at 11 locations in the West Indian Ocean

Country	Site	% assigned own site	1st migrant		2nd migrant	
			Most probable source	Other probable sources	Most probable source	Other probable sources
Mozambique	ZIS	94	Unknown			
Mozambique	ZIN	100				
Mozambique	ZP	88	Unknown		TZF	TZM
Tanzania ‐ Zanzibar	TZF	92	Unknown			
Tanzania ‐ Zanzibar	TZM	90	Unknown			
Tanzania ‐ Zanzibar	TZC	100				
Tanzania ‐ Zanzibar	TZN	93	TZC	KM		
Tanzania‐Mainland	TM	100				
Kenya	KM	94	Unknown			
Madagascar	MAE	100				
Madagascar	MAW	100				

Note

For each site (acronyms as in Table 1), the sampling sites are presented in rows and the percentage of individuals that is assigned to their own sampling site is shown (% assigned own site). A maximum of two migrants were found at one sampling site and the putative origin of each migrant is shown. When more than one location showed likelihoods above the threshold of 0.1, the most probable as well as other probable sources are shown. We calculated the probability that an individual belongs to the population from which it was sampled using a partially Bayesian criterion (Rannala & Mountain, 1997) and compared the likelihood of exclusion of an individual to a distribution of likelihoods of 10,000 simulated genotypes in order to define a statistical threshold (Paetkau, Slade, Burden, & Estoup, 2004; Underwood, Smith, Oppen, & Gilmour, 2007) with a type I error of 0.01. We excluded an individual from its sampling site when the probability for exclusion was above 95% and then excluded the migrants from the dataset, which served as the reference to which migrants were assigned. We assigned migrants to another sampled population when the probability was *p* ≥ 10% for this other population (Underwood et al., 2007).

The bottleneck tests indicated that TZC on Zanzibar and ZIS in Mozambique might have experienced a recent population decline (*p*‐value < 0.05 for TPM and SMM) and TZF on Zanzibar exhibited signs of a recent expansion (*p*‐value < 0.05 for TPM and SMM).

### 3.4 Isolation by distance

“Sea distance” ranged from ~15 to ~2,700 km. The two Madagascan sites are most geographically close, while the samples from Inhaca, Mozambique (ZIN and ZIS), and the Kenyan site (KM) are furthest apart. There was no evidence for a significant correlation between sea distance and the genetic distance measure *F*_ST_ (*p* = 0.17, *r = *0.14) nor *G*’_ST_ (*p* = 0.14, *r* = 0.17), but sea distance is significantly correlated with Jost's *D*_EST_ (*p* = 0.03, *r* = 0.37). Although most pairwise *F*_ST_ values are significant, *D*_EST_ is arguably the most meaningful measure for our dataset given that the observed heterozygosity is very low.

## 4 DISCUSSION

The overall aim of this study was to understand the large‐scale (1000s km) population genetic pattern of the seagrass *T. hemprichii* in the WIO region for regional conservation purposes. The study contributes to the knowledge of population genetic patterns of important species in the region, but also where in fact little is known on population genetic patterns in general. Genotyping of the seagrass *T. hemprichii* was successful, and we found four distinguished genetic clusters separated geographically on various spatial scales (10s–1000s km). Interestingly, a clear genetic cluster co‐occurs with a local system of eddies between the mainland of Tanzania and Zanzibar. Gene flow was directional and strongest from Madagascar toward the coasts of Kenya and western Zanzibar, spatially coinciding with the NEMC and the “continuing” EACC (Zavala‐Garay et al., 2015). Our findings indicate that the genetic structure of *T. hemprichii* in the WIO is influenced by large oceanic currents (SEC, NEMC, and EACC), as well as by local hydrodynamic patterns (Zanzibar channel). The study, therefore, emphasizes the necessity to consider different spatial scales to understand metapopulation dynamics and identify source populations of a habitat providing plant species.

### 4.1 Genetic structure and connectivity of *T. hemprichii* in the WIO

We show significant genetic structure among most of the sampled *T. hemprichii* meadows and find a relation between genetic differentiation (*D*_EST_) and increasing geographic distance. Nevertheless, large‐scale currents in the area seem to play an important role for the observed genetic differentiation and the four genetic clusters suggested by the TESS analysis conform well with the major oceanographic currents in the WIO. We hypothesized that the northbound SEC, NEMC, and EACC should influence the genetic structure from Madagascar in the South to Tanzania and Kenya in the North, leading to genetic uniformity. Indeed, the sites from the east coast of Zanzibar (TZN and TZC) and Kenya (KM) are not significantly differentiated from each other and show high admixture levels, indicating that they receive propagules from more southern sites. Madagascar also showed a large differentiation compared to the other sampling sites. As predicted based on the directionality of the SEC and NEMC, we found high asymmetric migration from Madagascar to Zanzibar (TZC in particular) and Kenya. The TESS analysis confirms gene flow from the Madagascan cluster (blue in the TESS analysis) to these two sites, and suggests additional gene flow to one more site on Zanzibar and one in Mozambique (Figure 2). Asymmetrical patterns of seagrass migration have also been found in a study on *T. hemprichii* in the Indo‐Australian archipelago, where eastern Indonesia was identified as an important source (Hernawan et al., 2016).

The Zanzibar channel cluster (sites TZF, TZM, and TM; green in the TESS analysis) is, however, clearly differentiated from all other sampling sites. The topography of the Zanzibar channel has a strong effect on the bottom current as it creates two gyres systems that turning clockwise and meet in front of the Zanzibar town. The gyres are caused by current systems that move to the north but differ in their velocity between the two revising monsoons. The gyres are stronger in the SE monsoon and are reduced in the NE monsoon, however, the circulation is all year around. In fact, eddies have been recorded in the Zanzibar channel, which could explain the lack of gene flow between these sites and that they are differentiated from all our other sampling sites. Similar patterns of genetic differentiation have also been observed for corals (Souter et al., 2009; van der Ven et al., 2016), and therefore it seems likely that the sites in the Zanzibar channel are subject to local oceanographic conditions that result in propagule retention and oceanographic isolation.

A higher admixture was found for the sites in Mozambique, Zanzibar, and Kenya, which is in concordance with other genetic studies in the area on crabs (Fratini, Ragionieri, & Cannicci, 2010), corals (van der Ven et al., 2016), and fish (Visram et al., 2010). The cluster in Mozambique is under the influence of the eddies in the Mozambique. Pemba (ZP) in the Mozambique channel has the highest number of polymorphic loci observed in our study, supporting the hypothesis of Obura (2012) that the Mozambique channel exhibits high species and genetic diversity for a number of taxa, because of high immigration with the NEMC and retention by the Mozambique channel eddies. The most geographically isolated populations were those at Inhaca Island, which were also genetically the most differentiated populations. These populations receive no gene flow from the other assessed sites and supply only few migrants to other sites.

Interestingly, a model‐based assessment of coral reef larvae dispersal in the WIO (Crochelet et al., 2016) found oceanographic clusters that are very similar to our genetic clusters. When assuming drift durations of 10–20 days, which are biologically realistic durations for *T. hemprichii*, the Madagascan sites are predicted to form one cluster, the south Mozambique sites another one, and all the remaining sites are predicted to be grouped into a third oceanographic cluster. The only genetic cluster that was not predicted by this hydrodynamic model was the one based on individuals from the Zanzibar channel, which is most likely because the spatial resolution of this large‐scale model does not fit the small‐scale detection level needed to show this separation. The findings by Crochelet et al. (2016) strongly confirm our hypothesis that the major hydrodynamic features shape population genetic structure of *T. hemprichii* in the WIO region. In the future, it would be valuable to examine historical, geographic, and environmental factors in depth as they may partly contribute to shaping the distribution of genetic variation of *T. hemprichii*. Indeed, a study on the seagrass *Z. capensis* studied in Southern and Eastern Africa could only detect population clustering when they included putative outlier loci, loci likely under selection driven by environmental factors, into their analysis (Phair et al., 2019).

### 4.2 Contribution of clones and heterozygote deficits

Asexual reproduction is common in seagrasses and has knock‐on effects on genetic variation of populations. We detected high genotypic richness at most sites, despite distances of 10–150 m between individuals, which suggests that both sexual and asexual reproduction modes are an important in *T. hemprichii* (mean *R* = 0.65). Only one site in Mozambique (ZIN) showed a dominant asexual reproduction mode. Significantly heterozygote deficits (positive *F*_IS_–values) were observed for all sampled populations, a finding commonly observed in seagrasses in general (Arnaud‐Haond, Stoeckel, & Bailleul, 2019) and found in *T. hemprichii* in the Indo‐Australian Archipelago as well (Hernawan et al., 2016). Heterozygote deficits may result from several factors including null alleles, inbreeding, linkage (LD), genetic patchiness at a local scale due to a mix of differentiated cohorts (Wahlund effect), partial clonality (Arnaud‐Haond et al., 2019), and recent admixture (Allendorf, Luikart, & Aitken, 2013; van Oppen, Lutz, De'ath, Peplow, & Kininmonth, 2008). In this study, inbreeding and/or lowly polymorphic microsatellites seem the most likely explanation for the high and significant *F*_IS_ values, as a study of the closely related *Thalassia testudinum* at two sites in Mexico found no prevalence of inbreeding (Van Tussenbroek et al., 2016). The microsatellite markers used in our study were developed for Indo‐Australian *T. hemprichii* (van Dijk et al., 2014) and the observed allelic richness and heterozygosity in our samples from the WIO were low compared to the *T. hemprichii* samples from the Indo‐Australian archipelago (Hernawan et al., 2016). The combination of low marker polymorphism and high clonality limits the power of the genetic inference analyses performed here (Hale, Burg, & Steeves, 2012; Ryman et al., 2006) and give evidence for the importance of developing markers that are more informative in the WIO. In particular, the combination of high clonality and low marker polymorphism found in this study results in a high likelihood that a large proportion of alleles are not detected (Hale et al., 2012), which has impacts on all F_ST_ related measures. As we observed very high values from the F_ST_ measures and low polymorphism, we used the approach suggested by Wang (2015) to test whether our estimates are substantially affected by mutations. Results (not shown) revealed no significant negative relationship between the two measures, indicating that results are reliable (Wang, 2015).

A previous study on the clown fish *Amphiprion akallopisos* showed higher genetic diversity in the East Indian Ocean (EIO) compared to the WIO, suggesting that this fish species originated in the EIO (Huyghe & Kochzius, 2017). Similarly, we hypothesize that the origin of *T. hemprichii* is in the EIO and Pacific and low genetic diversity may be explained by the fact that our sampling was performed at the edge of its distributional range. Furthermore, a number of studies have shown limited gene flow between the WIO and the EIO and Pacific, while others have shown low genetic diversity in the WIO (reviewed in Ridgway & Sampayo, 2005).

### 4.3 Management implications and conclusion

This study is the first large‐scale genetic study of a seagrass in the WIO and results indicate that dispersal of *T. hemprichii* is to a large extent influenced by predominant currents in the area. Nevertheless, traditional isolation by distance models—where genetic differentiation increases with geographical distance—also seems to play a role at the assessed spatial scales in the WIO, as do local hydrodynamic features. For future conservation planning, the information on genetic clusters and breaks, predominant directions of gene flow and genetic diversity should be used to ensure that connectivity is maintained in the WIO. A regional scale approach to seagrass conservation is recommended, including effective cross‐boundary management.

## CONFLICT OF INTEREST

None declared.

## AUTHOR CONTRIBUTIONS

LMN and MG conceived the idea; LMN secured funding and led the research project; field sampling were conducted by MG, LMN, MEA, SM, AH, and JM; analyses were conducted by MJ; initial analyses were conducted by JL and LMN; SM and MS developed the maps; MJ led the writing and all authors contributed throughout the whole process and agreed on the last version.

## Data Availability

The matrix of microsatellite genotypes is archived on Dryad (https://datadryad.org/). Data from Jahnke, M., Gullström, M., Larsson, J., Asplund, M. E., Mgeleka, S., Silas, M. O., Hoamby, A., Mahafina, J., Nordlund, L. M. (2019). Population genetic structure and connectivity of the seagrass *Thalassia hemprichii* in the Western Indian Ocean is influenced by predominant ocean currents, *Ecology and Evolution*, https://doi.org/10.5061/dryad.0hn97r5.
