# Supplementary material for: Population genetic structure and connectivity of the seagrass Thalassia hemprichii in the Western Indian Ocean is influenced by predominant ocean currents
Source: Ecol Evol. 2019 Jul 23;9(16):8953–64. doi: 10.1002/ece3.5420 (PMC6706205; doi:10.1002/ece3.5420)
Supplement: Supplementary file 1 [file ECE3-9-8953-s001.docx]

**Supporting information**

**Appendix S1** PCR conditions for the analysis of the 11 *Thalassia hemprechii* populations in the West Indian Ocean. Shown are the volumes of each ingredient added to each multiplex. MM is the master-mix provided by the Qiagen Type-IT kit. Volumes for primers are based on 20uM working solutions.

**THH-a**

|  |  | add µL |
| --- | --- | --- |
|  | 2xMM | 3.175 |
| FAM | THH-34 | 0.02 |
| blue | THH-34 | 0.02 |
| FAM | TH07 | 0.025 |
| blue | TH07 | 0.025 |
| FAM | THH-5 | 0.01 |
| blue | THH-5 | 0.01 |
| NED | TH37 | 0.05 |
| yellow | TH37 | 0.05 |
| NED | TH73 | 0.02 |
| yellow | TH73 | 0.02 |
| VIC | THH-15 | 0.03 |
| green | THH-15 | 0.03 |
| VIC | TH66 | 0.02 |
| green | TH66 | 0.02 |
|  | water | 1.725 |
|  | DNA | 1 |
|  | total | 6.25 |

**THH-b**

|  |  | add µL | |
| --- | --- | --- | --- |
|  | 2xMM | | 3.175 |
| FAM | TH43 | | 0.01 |
| blue | TH43 | | 0.01 |
| FAM | THH-8 | | 0.025 |
| blue | THH-8 | | 0.025 |
| NED | TH52 | | 0.01 |
| yellow | TH52 | | 0.01 |
| NED | THH-3 | | 0.03 |
| yellow | THH-3 | | 0.03 |
| VIC | TH34 | | 0.025 |
| green | TH34 | | 0.025 |
| VIC | THH-41 | | 0.025 |
| green | THH-41 | | 0.025 |
|  | water | | 1.825 |
|  | DNA | 1 | |
|  | total | 6.25 | |

**Appendix S2** Primer details for the analysis of the 11 *Thalasia hemprechii* populations in the West Indian Ocean.

| Name | F | R | Label for F | plex |
| --- | --- | --- | --- | --- |
| THH-3 | GGGAATTGCCTCAACTCATT | TTTGTTAAATCGCATTTGCTTC | NED | THH-b |
| THH-8 | GGACCTGAAGAGGGATGTGA | TCACATGCATACACAAACTGCAT | FAM | THH-b |
| THH-15 | CTCGCTAGGGGGATTGTGT | TCAACTAAAACAAAGGCAAGGAAA | Yakima Yellow | THH-a |
| THH-41 | GAACCGCATAGAAGTACAATCAAA | TCATGGTACATGCTCAGTCTAATG | Yakima Yellow | THH-b |
| TH07 | GTGGAGTTTGGGTTCTACTAAG | CAAACACAATTGCCAAGC | FAM | THH-a |
| TH34 | TTTCCATCTTCACAAACTCTT | ATAGGGAGAGGAGGAGTAGC | Yakima Yellow | THH-b |
| TH37 | GTGTGAGAGGAGAGTGAGGA | CTCACACGTAGTGACCCCT | NED | THH-a |
| TH43 | ACTTGGAAAACAGCTGGTAA | CGTGTGTGACCACAATCTAC | FAM | THH-b |
| TH52 | TCTCTAGGTTGGACGGCTAT | CCTTACCTTCGAATTAGGGT | NED | THH-b |
| TH66 | GCAAGAGATCAGGTCGATAA | GACCATTGAGGGTGATTATT | Yakima Yellow | THH-a |
| TH73 | TCACTACCTTGACTTAGGGC | GATTCCAACAATCCCTAACA | NED | THH-a |
| THH-5 | CACGAAATTGCAACCAACAC | TTTGTTGGATCGCTATGAGTATG | FAM | THH-a |
| THH-34 | TGGAGATGAGGACAGGTGTG | CGCTACCCTCGGTTTATTCC | FAM | THH-a |

**Appendix S3** *F_ST_* (lower diagonal) and *G’_ST_* (upper diagonal) for the of the 11 *Thalassia hemprechii* populations in the West Indian Ocean. Significant (based on confidence intervals) pairwise genetic differentiation values are shown in bold. Site acronyms as in Table 1 of the main manuscript.

| **Pops** | **ZIS** | **ZIN** | **ZP** | **TZF** | **TZM** | **TZC** | **KM** | **TZN** | **MAE** | **MAW** | **TM** |
| --- | --- | --- | --- | --- | --- | --- | --- | --- | --- | --- | --- |
| **ZIS** | 0 | 0.02 | 0.06 | **0.66** | **0.70** | **0.26** | **0.22** | **0.30** | 0.16 | **0.48** | **0.51** |
| **ZIN** | -0.02 | 0 | 0.21 | **0.61** | **0.66** | **0.33** | **0.27** | **0.41** | **0.30** | **0.53** | **0.45** |
| **ZP** | 0.03 | 0.13 | 0 | **0.59** | **0.64** | 0.14 | 0.11 | 0.11 | 0.01 | **0.30** | **0.46** |
| **TZF** | **0.53** | **0.51** | **0.47** | 0 | -0.04 | **0.42** | **0.39** | **0.50** | **0.67** | **0.60** | **0.47** |
| **TZM** | **0.56** | **0.56** | **0.51** | -0.05 | 0 | **0.46** | **0.44** | **0.56** | **0.73** | **0.68** | **0.53** |
| **TZC** | **0.19** | **0.23** | 0.09 | **0.31** | **0.34** | 0 | -0,01 | 0.19 | **0.26** | **0.42** | **0.41** |
| **KM** | **0.15** | **0.17** | 0.06 | **0.30** | **0.32** | -0.03 | 0 | 0.16 | 0.19 | **0.33** | **0.37** |
| **TZN** | **0.23** | **0.33** | 0.07 | **0.41** | **0.47** | **0.13** | **0.11** | 0 | 0.17 | 0.14 | **0.48** |
| **MAE** | 0.08 | **0.19** | -0.05 | **0.57** | **0.64** | **0.15** | 0.09 | 0.10 | 0 | 0.30 | **0.51** |
| **MAW** | **0.33** | **0.41** | **0.18** | **0.49** | **0.60** | **0.26** | **0.19** | 0.06 | 0.21 | 0 | **0.58** |
| **TM** | **0.38** | **0.33** | **0.33** | **0.39** | **0.46** | **0.28** | **0.24** | **0.38** | **0.41** | **0.50** | 0 |

**Appendix S4** Jost *D_EST_* for the of the 11 *Thalassia hemprechii* populations in the West Indian Ocean. Significant (based on confidence intervals) pairwise genetic differentiation values are shown in bold**.** Site acronyms as in Table 1 of the main manuscript.

| **Pops** | **ZIS** | **ZIN** | **ZP** | **TZF** | **TZM** | **TZC** | **KM** | **TZN** | **MAE** | **MAW** | **TM** |
| --- | --- | --- | --- | --- | --- | --- | --- | --- | --- | --- | --- |
| **ZIS** | 0 |  |  |  |  |  |  |  |  |  |  |
| **ZIN** | 0.00 | 0 |  |  |  |  |  |  |  |  |  |
| **ZP** | 0.00 | 0.01 | 0 |  |  |  |  |  |  |  |  |
| **TZF** | **0.08** | **0.08** | **0.06** | 0 |  |  |  |  |  |  |  |
| **TZM** | **0.09** | **0.09** | **0.066** | 0 | 0 |  |  |  |  |  |  |
| **TZC** | **0.03** | **0.05** | 0.01 | **0.05** | **0.05** | 0 |  |  |  |  |  |
| **KM** | **0.03** | **0.04** | 0.01 | **0.04** | **0.05** | 0.00 | 0 |  |  |  |  |
| **TZN** | **0.03** | **0.04** | 0.00 | **0.04** | **0.04** | 0.01 | 0.01 | 0 |  |  |  |
| **MAE** | 0.01 | 0.01 | 0 | **0.05** | **0.06** | **0.03** | 0.01 | 0.00 | 0 |  |  |
| **MAW** | **0.05** | 0.05 | 0.02 | 0.03 | 0.03 | 0.03 | 0.02 | 0.00 | 0.01 | 0 |  |
| **TM** | **0.06** | **0.07** | **0.04** | **0.01** | **0.01** | **0.05** | **0.04** | **0.05** | **0.04** | 0.03 | 0 |

**Appendix S5** DIC and deltaK analysis of TESS and STRUCTURE. Left: Average DIC (Deviance Information Criteria) of the TESS analyses that points to *K*_max_ = 4 as the most likely number of clusters. The minimal DIC is at K = 7. Right: deltaK analysis of the Structure analyses that points to K = 2 as the most likely number of clusters, with secondary peaks at K = 4 and K = 7.

**
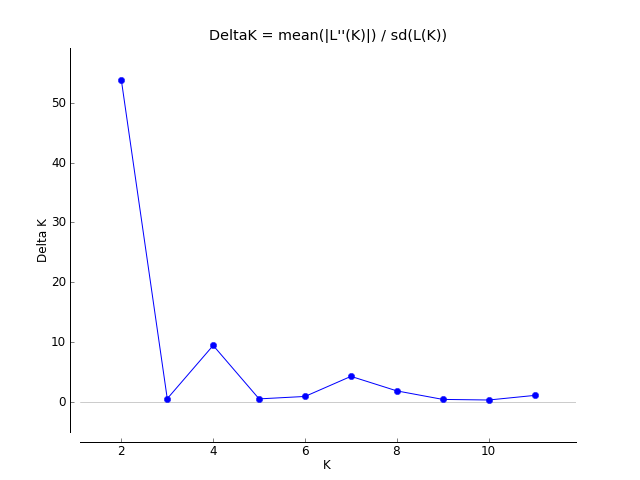
**

**Appendix S6** TESS and STRUCTURE clustering analyses for the 11 *Thalassia hemprechii* populations in the West Indian Ocean. Shown are the merged outputs for *K/K*_max_ =2-7. Within each plot, each vertical bar represents an individual belonging to the sampling location indicated under the x-axis, clusters are colour coded, and the y-axis of each plot shows the proportion of the genotype belonging to each cluster. Site acronyms as in Table 1 of the main manuscript.

**
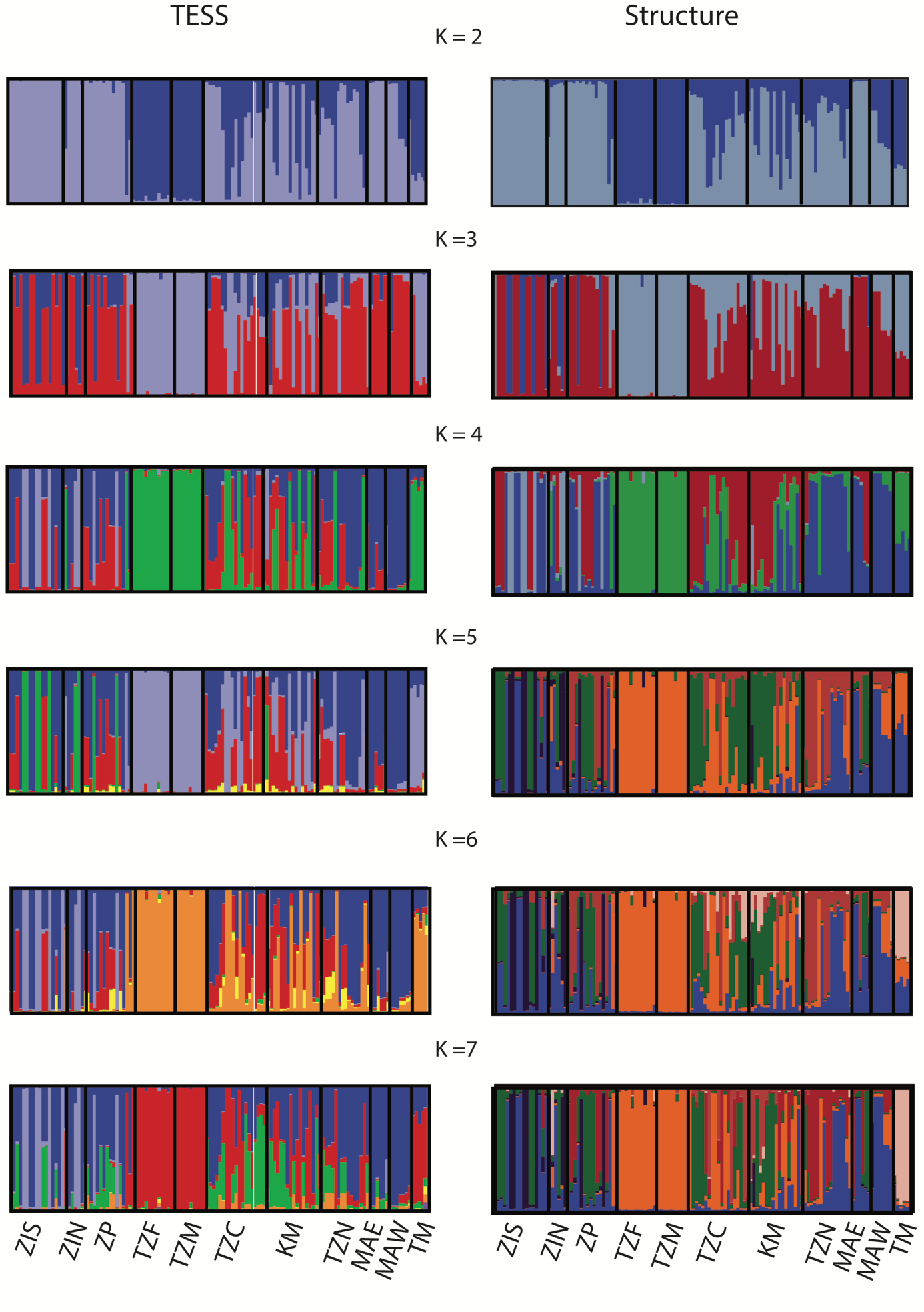
**
